# Evaluation of Rapid Antigen Detection Kits for Detection of SARS-CoV-2 B.1.1.529

**DOI:** 10.1007/s44229-022-00009-8

**Published:** 2022-05-31

**Authors:** Gannon C. K. Mak, Stephen S. Y. Lau, Kitty K. Y. Wong, C. S. Lau, Edman T. K. Lam, Ken H. L. Ng, Rickjason C. W. Chan

**Affiliations:** Microbiology Division, Public Health Laboratory Services Branch, Centre for Health Protection, Department of Health, 9/F, Public Health Laboratory Centre, 382 Nam Cheong Street, Shek Kip Mei, Kowloon, Hong Kong, Special Administrative Region China

**Keywords:** SARS-CoV-2, COVID-19, Rapid antigen detection, Omicron, B.1.1.529

## Abstract

**Background:**

Currently, there is a lack of studies evaluating rapid antigen detection (RAD) kits to detect SARS-CoV-2 B.1.1.529.

**Objective:**

To evaluate the analytical sensitivity of seven RAD kits to detect SARS-CoV-2 B.1.1.529.

**Study design:**

The analytical sensitivity was determined by means of limit of detection (LOD). A dilution set using a respiratory specimen collected from a COVID-19 patient infected with SARS-CoV-2 B.1.1.529 was prepared. RT-PCR was used as a reference method.

**Results:**

The LOD results showed that all seven RAD kits had comparable analytical sensitivity for detection of SARS-CoV-2 B.1.1.529.

**Conclusions:**

The RAD kits selected in the current study may be used for first-line screening of the recently emerged SARS-CoV-2 B.1.1.529.

## Introduction

Severe acute respiratory syndrome coronavirus 2 (SARS-CoV-2) is continuously evolving. The World Health Organization (WHO) classified those SARS-CoV-2 variants having global public health impacts as variants of concern (VOCs). Currently, there are four VOCs, namely, B.1.1.7, B.1.351, P.1, B.1.617.2, and B.1.1.529, according to the Phylogenetic Assignment of Named Global Outbreak (PANGO) nomenclature system [1]. The latest VOC was SARS-CoV-2 B.1.1.529; it was classified as a VOC and named Omicron by the WHO on November 26, 2021 [2]. Since then, many countries have reported detection of SARS-CoV-2 B.1.1.529, and it has become the dominant variant in countries such as the United Kingdom and United States [3, 4].

The gold standard for detection of SARS-CoV-2 is RT-PCR. Rapid antigen detection (RAD) kits are an alternative to RT-PCR due to the fast results and ease of use, although these kits are inferior to RT-PCR in terms of sensitivity. Different groups have compared the sensitivity of RAD kits in detecting different SARS-CoV-2 VOCs [5–8]. However, these studies were lacking in data against SARS-CoV-2 B.1.1.529. The current study assessed the performance of RAD kits in terms of limit of detection (LOD) using a dilution set of a clinical specimen.

## Methods

### Respiratory Specimen

We used a respiratory specimen, combined nasopharyngeal swab and throat swab, throughout this evaluation. It was collected from the first SARS-CoV-2 B.1.1.529 case detected in Hong Kong on November 15, 2021 [9]. This specimen had sufficient quantity and high enough viral load to fulfil the criteria for this evaluation.

### SARS-CoV-2 RAD Kits

In April 2020, the WHO designated the Public Health Laboratory Services Branch (PHLSB) as one of the WHO COVID-19 reference laboratories [10]. We routinely reviewed and evaluated RAD kits that were introduced to our laboratory by local suppliers [11–16]. We selected seven RAD kits for the current study, based on availability and past satisfactory performances. A summary of the seven RAD kits is shown in Table 1. For ease of communication, the kits were coded arbitrary from N1 to N7. We procured three of the kits, N1 to N3. The remaining four kits, N4 to N7, were gifted to our laboratory by local suppliers from December 2020 to June 2021 for this evaluation.

**Table 1:** Rapid antigen detection kits evaluated in the present study

Kit	Manufacturer	Country of manufacturer	Code^a^	Sample type	Target	Turnaround time
Roche SARS-CoV-2 Rapid Antigen Test	SD BIOSENSOR	Republic of Korea	N1	Nasopharyngeal swab, specimens in transport media	Nucleocapsid	15–30 min
Panbio COVID-19 Antigen SELF-TEST	Abbott Rapid Diagnostics Jean GmbH	Germany	N2	Nasal swab	Nucleocapsid	15–20 min
INDICAID COVID-19 Rapid Antigen Test	PHASE Scientific International Ltd	Hong Kong SAR	N3	Nasal swab, nasopharyngeal swab	Not specified	20–25 min
Rapid SARS-COV-2 Antigen Test Card	Ximan Boson Biotech Co., Ltd	China	N4	Nasopharyngeal swab	Nucleocapsid	15–20 min
Rapid SARS-CoV-2 Antigen Test Card	MP Biomedicals Germany GmbH	Germany	N5	Nasal swab, nasopharyngeal swab, oropharyngeal swab	Nucleocapsid	15–20 min
COVID-19 Antigen Saliva Test	ulti med Products (Deutschland) GmbH	Germany	N6	Saliva	Nucleocapsid	10 min
SARS-CoV-2 Virus Antigen Detection Kit	BGI PathoGenesis Pharmaceutical Technology Co., Ltd	China	N7	Nasal swab	Not specified	15–20 min

This table was prepared by retrieving information from the product inserts delivered together with the kits

^a^For ease of communication throughout the article, each kit was assigned a code from N1 to N7

The kits selected were based on lateral flow principles. SARS-CoV-2 antibody was applied as an immobilized coating on the test cassettes to detect viral antigen. The test results could be interpreted by naked eyes. The test results were assessed and read by two technicians. Results grading of the band intensity were based on a previous study [7]. In case of disagreement, a third technician interpreted the test results.

### LOD of RAD Kits

We used LOD to assess the analytical sensitivity of RAD kits. We prepared the dilution set by performing serial tenfold dilution using viral transport medium (VTM). We prepared the VTM ourselves, and our previous results showed that there were no effects on the sensitivity of the RAD kits [14].

We used a modified sample processing method in this study that was consistent with our previous studies [13, 14]. In brief, we mixed a 350 μl specimen with the kit’s extraction buffer/diluent. For the N6 kit, we added the 350 μl specimen directly to the test cassette since the extraction buffer/diluent was not available. We performed other procedures according to the manufacturer’s instructions. We only performed one replicate for each dilution point due to the lack of samples and low quantity of kits.

We used real-time RT-PCR cycle threshold (Ct) value to estimate the viral concentrations of each dilution point [11]. We measured each dilution point in duplicate. We then recorded the Ct values for the average of two runs.

## Results

Table 2 summarizes the LOD results for RAD kits against SARS-CoV-2 B.1.1.529. All kits could detect dilution point 10^−2^, and the corresponding Ct value was 23.82. Three RAD kits could detect dilution point 10^−3^, and the corresponding Ct value was 27.08.

**Table 2:** Comparison of limit of detection for seven rapid antigen detection kits to detect SARS-CoV-2 B.1.1.529

Dilution	RT-PCR	Rapid antigen detection kits^a^						
		N01	N02	N03	N04	N05	N06	N07
10^−1^	20.37	+ + +	+ + +	+ + +	+ + +	+ + +	+ + +	+ + +
10^−2^	23.82	+	+ +	+ +	+	+ +	+ +	+ +
10^−3^	27.08	−	+	+	−	+	−	−
10^−4^	30.81	−	−	−	−	−	−	−
10^−5^	33.39	ND	ND	ND	ND	ND	ND	ND
10^−6^	−	ND	ND	ND	ND	ND	ND	ND

The RT-PCR and RAD results were based on testing the serial tenfold dilution of the respiratory specimen, combined nasopharyngeal swab and throat swab, obtained from the first SARS-CoV-2 B.1.1.529 case diagnosed in Hong Kong on November 15, 2021

+++ strong positive, ++ positive, + weak positive, − negative; *ND* not done

^a^The details of each kit are summarized in Table 1

The LOD for RT-PCR was 10^−5^; the results were concordant with our previous studies showing that RT-PCR was at least 100-fold more sensitive than the RAD kits against non-VOC strains [12–15].

## Discussion

In this study, our results showed that different RAD kits had similar analytical sensitivity for detection of SARS-CoV-2 B.1.1.529. We employed similar methods to those previously used for evaluating RAD kits; thus, variation in other parameters such as specimen input volume and viral load quantification could be minimized [11–15].

SARS-CoV-2 VOCs are characterized by the S protein mutations. Most RAD kits target SARS-CoV-2 N protein. Unlike RT-PCR assays, the performance of different RT-PCR assays can be checked by aligning the sequences of primers and probes against SARS-CoV-2 viruses. It was impossible to check the performance of RAD kits against different SARS-CoV-2 viruses, and information regarding antibodies used for RAD kits was not available. In the present study, the N protein of the SARS-CoV-2 B.1.1.529 strain (GISAID accession: EPI_ISL_6590782) showed four mutations/changes, namely P13L, 31–33 deletion of ERS, R203K, and G204R, compared with the reference strain WIV04 (EPI_ISL_402124). Our data showed that these changes did not significantly affect the effectiveness of RAD kits evaluated in the present study.

The main objective of the current study was to determine if the commercially available RAD kits are capable of detecting SARS-CoV-2 B.1.1.529. The accurate and precise ranking of RAD kits was not our primary focus. It would have been ideal to assess more dilution points such as 1:2 and 1:5 between the two serial tenfold dilution points. In addition, each dilution point should be performed in replicates. Given that an extra quantity of specimens and RAD kits were required, in addition to the limited manpower and resources, we determined that a serial tenfold dilution and single replicate were sufficient. It is expected that RAD kits share a similar performance within a tenfold difference based on this study design.

This study had several limitations. First, we only used one SARS-CoV-2 B.1.1.529 virus to assess analytical sensitivity. Second, we only measured the LOD of each RAD kit. Although analytical sensitivity does not reflect clinical sensitivity, our previous studies showed that analytical sensitivity correlated well with clinical sensitivity [11–13, 15]. In addition, all of the RAD kits evaluated in this study could detect concentration Ct 23.82, which was in accordance with a recent review summarizing 24 studies worldwide [17]. The LOD results enable us to assess RAD kits quickly when numerous kits are evaluated. Therefore, our results showed that the RAD kits used in this study may be used for first-line screening of SARS-CoV-2 B.1.1.529 cases. Finally, we did not test the specificity of the RAD kits. However, this issue was not a major concern in view of the currently evaluated RAD kits [18].

## Conclusion

The evaluation results of different RAD kits are important to help us implement the test appropriately. Due to the emergence of different SARS-CoV-2 variants as well as the latest developments in RAD kits, the performance of RAD kits should be regularly monitored so that guidance can be provided to different clinical settings.

## Abbreviations

Ct: Cycle threshold
LOD: Limit of detection
RAD: Rapid antigen detection
VOC: Variant of concern

## Declarations

### Availability of data and material

Data sharing is not applicable to the current study as the data are comprehensively described throughout this article.
